# A Systematic Literature Review on Cyber Threat Intelligence for Organizational Cybersecurity Resilience

**DOI:** 10.3390/s23167273

**Published:** 2023-08-19

**Authors:** Saqib Saeed, Sarah A. Suayyid, Manal S. Al-Ghamdi, Hayfa Al-Muhaisen, Abdullah M. Almuhaideb

**Affiliations:** 1 SAUDI ARAMCO Cybersecurity Chair, Department of Computer Information Systems, College of Computer Science and Information Technology, Imam Abdulrahman Bin Faisal University, P.O. Box 1982, Dammam 31441, Saudi Arabia; 2 Department of Computer Information Systems, College of Computer Science and Information Technology, Imam Abdulrahman Bin Faisal University, P.O. Box 1982, Dammam 31441, Saudi Arabia2230500091@iau.edu.sa (H.A.-M.); 3 SAUDI ARAMCO Cybersecurity Chair, Department of Networks and Communications, College of Computer Science and Information Technology, Imam Abdulrahman Bin Faisal University, P.O. Box 1982, Dammam 31441, Saudi Arabia

**Keywords:** cybersecurity, cyber threat intelligence, business organizations, mitigation

## Abstract

Cybersecurity is a significant concern for businesses worldwide, as cybercriminals target business data and system resources. Cyber threat intelligence (CTI) enhances organizational cybersecurity resilience by obtaining, processing, evaluating, and disseminating information about potential risks and opportunities inside the cyber domain. This research investigates how companies can employ CTI to improve their precautionary measures against security breaches. The study follows a systematic review methodology, including selecting primary studies based on specific criteria and quality valuation of the selected papers. As a result, a comprehensive framework is proposed for implementing CTI in organizations. The proposed framework is comprised of a knowledge base, detection models, and visualization dashboards. The detection model layer consists of behavior-based, signature-based, and anomaly-based detection. In contrast, the knowledge base layer contains information resources on possible threats, vulnerabilities, and dangers to key assets. The visualization dashboard layer provides an overview of key metrics related to cyber threats, such as an organizational risk meter, the number of attacks detected, types of attacks, and their severity level. This relevant systematic study also provides insight for future studies, such as how organizations can tailor their approach to their needs and resources to facilitate more effective collaboration between stakeholders while navigating legal/regulatory constraints related to information sharing.

## 1. Introduction

Cybersecurity is a significant concern for businesses worldwide, because cyber attackers constantly target corporate data and information technology (IT) resources to make money or gain a geopolitical advantage [1]. Cybersecurity can be defined as securing individual or organizational electronic data from unauthorized access. An attempt to gain unauthorized access is termed a cyber-attack, and these attacks may entail the theft of private data, intellectual property, confidential business strategy plans, and/or the disruption of mission-critical IT systems. Organized crime syndicates and nation-state paramilitary cyber organizations have also started using cyber-attacks as an operational strategy that has led to the development of advanced persistent threats (APTs), which are becoming increasingly difficult for organizations to defend against despite having formalized cybersecurity systems [2,3].

Cyber threat intelligence (CTI) has emerged as a potential solution for businesses to address security events’ increasing quantity and complexity. CTI refers to the proactive identification and analysis of cyber threats. However, subscribing to different threat intelligence sources can lead to information overload. A Threat Intelligence-Sharing Platform (TISP) can take cyber threat information data and turn it into actionable intelligence that can be fed into multiple technologies to help in incident response. Information security firms and the ecosystem currently offer TISP solutions in two categories: content aggregation, which provides numerous threat data feeds, and threat intelligence management, which generates economic value from the data obtained [4].

CTI is a process of gathering, analyzing, and distributing information to identify, monitor, and anticipate cyber threats. CTI can help businesses become more proactive in cybersecurity by identifying vulnerabilities before attackers exploit them [5]. For example, suppose a particular threat actor has been known to target companies using a specific malware or attack method. In this case, CTI could help to identify this pattern early on so that intrusion detection systems can be assigned to look for those patterns specifically. CTI also plays an essential role in detecting attacks by setting intrusion detection systems based on practices associated with certain threat actors or types of attacks identified by analyzing gathered intelligence data. Furthermore, CTI provides specific security plans tailored toward countering the mannerisms used by cyber threat actors, making it an essential tool for organizations looking at preventing, detecting, and responding effectively against potential cyber-attacks [2].

As a response to the benefits it provides and the present market trend, CTI has attracted the attention of most organizations. Consequently, CTI alters the organization’s processes and actions as it faces various issues [6,7]. Therefore, the current research will document state-of-the-art cyber threat intelligence. We have conducted a systematic literature review based on the scientific literature published in the last five years to highlight how evolving procedures and technology have helped organizations to improve the cybersecurity of their critical infrastructures by improving CTI. Based on the review, we have outlined a layered CTI framework for organizations to improve their cybersecurity resilience. This model provides a starting point for other researchers to deploy and test applications in organizations to improve their CTI.

This paper’s structure is as follows: Section 2 defines the procedures for selecting primary studies for systematic analysis. Section 3 explains the results of all of the designated significant research. Section 4 considers the findings in connection to the previously indicated research subjects. Section 5 concludes the analysis and makes some recommendations for further study.

## 2. Materials and Methods

Our review followed the guidelines provided by Kitchenham and Charters [8], along with Preferred Reporting Items for Systematic Reviews and Meta-Analyses (PRISMA) [9] recommendations for systematic review. An extensive systematic literature review (SLR) was conducted through an iterative planning, examination, and documentation process. Primary studies were extracted by directing keywords to scientific repositories. Keywords were designated to aid in developing study outcomes that contribute to the research. We mainly used “Cyber Threat” as the search term to extract relevant papers from the Institute of Electrical and Electronics Engineering (IEEE) digital library [10], the Association of Computing Machinery (ACM) digital library [11], and the university library’s online repository. Searches were performed on paper titles during March 2023. The inclusion criteria were as follows:

- The selected paper should focus on CTI in business organizations.
- The paper should be published between 2019 and 2023.
- All papers were journal articles or conference proceedings; any other publication type was excluded.

From the first database keyword searches, we identified 294 research works, of which eight were duplicates. After we checked the title and keywords on each paper under the inclusion/exclusion criteria, we found that 70 papers were not journal or conference publications. Also, 153 papers could not be retrieved. As a result, the number of papers available to review was left at 63. After reading the 63 articles in full and using the inclusion/exclusion criteria, we settled on 52 papers for inclusion in our study. To verify that extracted data passed an assessment of quality, they underwent a test to assess that the data were complete and relevant, and subsequently, this assessment to determine the accuracy of the information included in these studies. The process of identifying the extracted studies went through several stages to have a group of papers that passed the following quality assessment stages.

- Stage 1: The manuscript must be published in peer-reviewed journals or conference proceedings. Poster presentations, books, and blogs were left out due to quality concerns.
- Stage 2: The paper must be focused on the CTI domain that impacts organizations’ performance.
- Stage 3: The paper must be a case study, system application, or modeling implementation.

**Figure 1:**
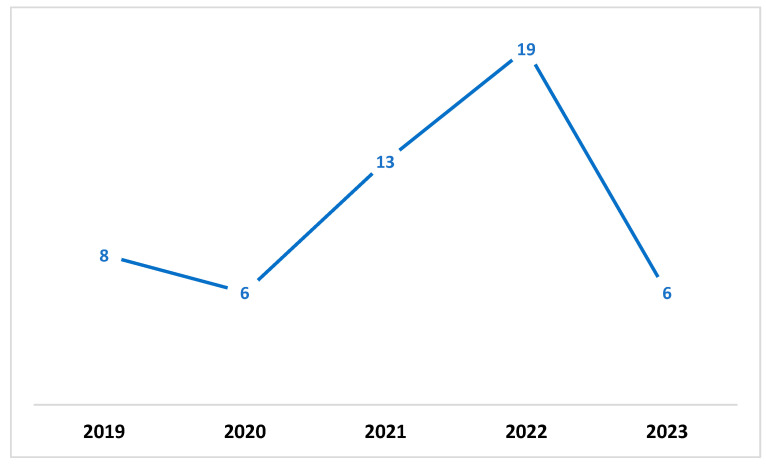
Annual total of original research articles.

**Figure 2:**
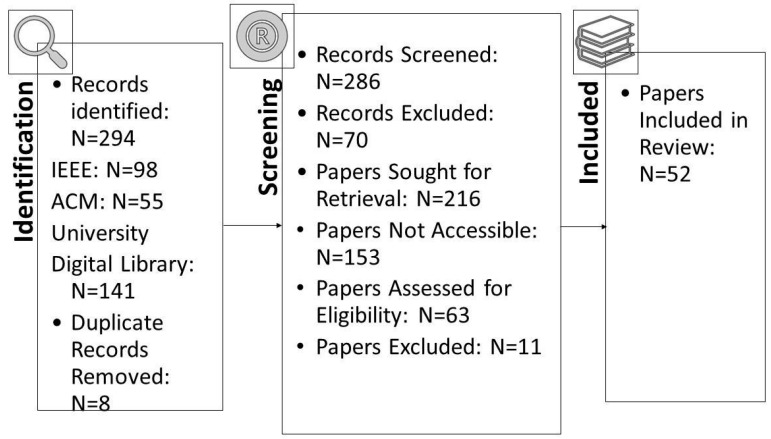
Number of accepted articles in SLR.

A quality evaluation checklist was used to evaluate the papers that were found. Eleven studies were excluded from the analysis, because they did not meet the checklist elements’ criteria. The information and data extracted from each paper were stored in a table containing important information for the classification of each paper. Figure 1 shows the distribution of the publication year of the paper. In contrast, Figure 2 presents the total number of accepted articles that went through the review process, starting from the selection of keywords in the selected databases to the last stage.

## 3. Results

The topics of the primary investigations were categorized more broadly based on the narrower emphasis of each publication. The Detection Model subcategory includes research to improve methods for detecting assaults, introduce novel detection models, or use novel detection characteristics. Knowledge Sharing and Training is an umbrella area for research on information dissemination, public education, and better decision making. Based on the presented categories, further analysis was conducted to extract each paper’s main details. 

### 3.1. Detection Model

Suryotrisongko et al. [12] developed an automated mechanism for detecting botnet Domain Generation Algorithm (DGA) attacks using natural language processing methods and machine-learning procedures. The authors developed a new model that identifies features from unstructured reports and determines cyber threat actors, achieving high accuracy rates of 96% and a precision rate of 96.4%. The model is based on query logs from Domain Name System (DNS) servers from 2004 to 2015, and it identified 107 malicious domain names associated with botnet traffic [12]. In another study, Moraliyage et al. [13] proposed using Artificial Intelligence (AI) to categorize the sites based on their content, using a new approach called explainable deep learning. The explainable deep-learning approach analyzes images and text on each site with advanced AI algorithms, such as the Convolutional Neural Network for image analysis with Gradient-weighted Class Activation Mapping (Grad-CAM) and pre-trained word embedding for text analysis. Combining these techniques in two learning pathways—one focused on images, and one focused on text—the method can accurately identify different types of onion services while explaining how it made those classifications based on specific features or patterns found within each site’s content [13].

In another study, Irshad and Siddiqui [14] proposed a mechanism to identify features from reports about cyber attackers. They used natural language processing methods and machine-learning algorithms, specifically an embedding model called “Attack2vec”. This model was trained on domain-specific embeddings and optimized for the specific language used in cybersecurity reports. The authors then used machine-learning algorithms to classify different cyber threat actors based on these extracted features. The suggested technique yields excellent accuracy rates of 96 percent accuracy, 96.4 percent precision, 95.58 percent recall, and 95.75 percent F1 measure (which combines precision and recall) [14].

Finding and deriving threat actions from unstructured CTI information is a difficult task. The current methods rely on semantic dependency and ontology, but they have limitations in accurately extracting all key threat actions and measuring their information content. Zhang et al. [15] proposed a new approach called EX-Action to address this issue. It is a multimodal learning approach that uses mutual information and natural language processing (NLP) methods to classify threat actions from unstructured CTI records. The framework consists of two main steps: 1. Threat actions are extracted by matching syntactic rules based on the sentence structure in the CTI report. 2. Extracted threats are identified using multimodal learning algorithms. Normalized mutual information (NMI) is used as an evaluation indicator to evaluate the completeness of extracted information content. The proposed method was tested on 243 unstructured CTI reports, with excellent accuracy reaching 79 percent [15].

In addition, the significance of cyber-physical systems is critical in developing a maintainable computing ecosystem for scalable and secure network design. Cha et al. [16] proposed a methodology that involved collecting data from network devices, extracting meaningful information such as file hash values and IP addresses, and distributing this information with a centralized institution called a cloud server (CS) to build a credible dataset. Duplicate data generated by multiple feeds were removed when the authors created their datasets. Blockchain technology was used to protect the integrity of CS’s centralized data and rewarded companies that contributed to creating trustworthy datasets. This system reduced network load while ensuring reliability, privacy, scalability, and sustainability for large-scale IoT systems that generate big data communication efficiently. In confined test settings, employing the IP addresses of open-source intelligence CTI feeds saved roughly 15% of storage space relative to total network resources [16].

Gong and Lee [17] proposed a framework intended to assist enterprises in real-time detection, analysis, and response to cyber threats and reduce the effect of cyber-attacks on business operations. The framework was comprised of four stages: threat intelligence collection, threat analysis and triage, incident response planning, and execution. The research provides a complete account of each step and discusses the tools and techniques that can be used to implement them. Furthermore, the study’s authors conducted several evaluation experiments on the effectiveness of the proposed framework in detecting and responding to cyber threats in an energy cloud platform. The results showed that the framework could detect and respond to cyber threats in real time and significantly reduce the time taken to detect and mitigate cyber-attacks. Overall, the CTI framework proposed in this study offers a comprehensive approach to incident response in an energy cloud platform. The framework can assist firms in proactively detecting and responding to cyber-attacks, lowering the risk of disruption to their operations, and improving the overall security posture of the energy cloud platform. The study also highlights several challenges related to CTI. One of the main challenges is the difficulty in obtaining high-quality intelligence relevant to the organization’s specific needs. Developing artificial intelligence requires significant resources and expertise to collect, analyze, and validate intelligence data. Another challenge is the lack of standardization in intelligence collection, analysis, and sharing, which makes it difficult to compare and evaluate intelligence from different sources. Additionally, the study highlights the need for organizations to balance the benefits of sharing intelligence with the risks of sharing sensitive information with third parties. Finally, the fast speed of technological progress and the changing character of cyber threats mean that organizations must continually adapt and update their CTI strategies to stay ahead of the threats [17].

Ejaz et al. [18] explored the applications of machine learning (ML) in visualizing patterns in CTI data to improve cybersecurity. The study highlights the importance of CTI in protecting organizations against cyber threats and using ML techniques to analyze and visualize large volumes of CTI data. Organizations can take proactive measures to protect their systems from potential attacks by identifying patterns in the data. The article also identified several challenges related to CTI, including the complexity and volume of data, the lack of standardization in data collection and sharing, and the need for skilled personnel to analyze and interpret the data. The study suggests that addressing these issues could enhance the effectiveness of CTI and cybersecurity measures. Overall, the article focuses on the potential of ML techniques to improve the visualization and analysis of CTI data and strengthen cybersecurity measures in organizations. There are several challenges related to CTI, including the fact that the quality of the data used to generate CTI can vary widely. Incomplete or inaccurate data can lead to flawed threat assessments and ineffective security measures. Additionally, information overload, or the volume of data related to cybersecurity threats, can be overwhelming, creating difficulties for organizations in identifying and prioritizing the most relevant threats. Also, practical CTI requires specialized skills and knowledge, which can be difficult for organizations to acquire and maintain. Collaboration and information sharing about cyber threats across organizations can be challenging, as it requires trust and cooperation between different entities. Building and maintaining a robust CTI capability can be expensive, particularly for smaller organizations with limited resources [18].

Mendez Mena and Yang [19] developed a framework for decentralized threat intelligence that can be applied to traditional networks and the Internet of Things (IoT). The authors argue that traditional centralized threat intelligence approaches are insufficient for the rapidly evolving threat landscape and the growing number of connected IoT devices. The proposed framework includes several components: distributed threat data collection and analysis, decentralized threat intelligence sharing, and autonomous threat response. The article discusses the advantages of a decentralized approach to threat intelligence, including improved scalability, resiliency, and privacy. The authors also highlight the challenges that must be addressed, such as ensuring trust and consensus among distributed nodes and addressing potential performance bottlenecks. Overall, the article provides a comprehensive overview of the proposed framework for decentralized threat intelligence and its potential applications in securing networks and IoT devices [19].

Liu et al. [20] highlighted how current threat intelligence systems rely heavily on manual analysis, which is time-consuming and prone to errors. Therefore, they proposed a machine-learning-based system that automatically identifies relevant threat intelligence and provides actionable insights. The proposed approach, TriCTI, uses a combination of neural networks and trigger detection algorithms to identify patterns in threat intelligence data. The system was trained on a large dataset of CTI reports and could automatically extract relevant indicators of compromise (IOCs) and identify potential threat actors. The authors evaluate TriCTI’s performance on several datasets and compare it to other state-of-the-art threat intelligence systems. They show that TriCTI outperforms other systems regarding precision and recall, indicating that it can effectively identify relevant threat intelligence. The article also discusses the potential applications of TriCTI in cybersecurity operations, such as incident response and threat hunting. The authors argue that TriCTI can significantly reduce the time and effort required to identify and respond to cyber threats. Overall, the article presents a comprehensive overview of TriCTI, a machine-learning-based system for CTI discovery [20].

Kiwia et al. [21] presented a taxonomy for banking trojans based on the cyber kill chain model, a framework used to describe the stages of a cyber-attack. The taxonomy structures the characteristics and behavior of banking trojans and develops more effective countermeasures. The authors use an evolutionary computational intelligence approach to identify the standard features and behavior of banking trojans and cluster them into different categories based on the stages of the cyber kill chain. The resulting taxonomy is comprised of six categories: intelligence gathering, weaponization, delivery, exploitation, installation, and command and control. The study also analyzes the characteristics and behavior of each category of a banking trojan, including the techniques they use to evade detection and spread, the types of information they target, and the impact they can have on the victims. The authors argue that understanding the behavior and characteristics of banking trojans is crucial for developing effective countermeasures to protect against these types of attacks. Overall, this study provides a valuable framework for understanding the behavior and characteristics of banking trojans and highlights the need for ongoing research and development of countermeasures to protect against these attacks [21].

Gong and Lee [22] presented the BLOCIS framework, which addresses the limitations of existing CTI-sharing systems, such as the vulnerability to Sybil attacks and the lack of privacy and accountability. The authors use a combination of blockchain technology and game theory to create a decentralized and trustless system for sharing CTI. The system allows participants to contribute threat intelligence anonymously while ensuring that other participants validate and verify the information before being added to the blockchain. The study also provides a detailed analysis of the BLOCIS framework, including its architecture, algorithms, and protocols. The authors evaluate the framework’s effectiveness using simulation experiments and compare it to existing CTI-sharing systems. The study results show that the BLOCIS framework effectively prevents Sybil attacks and ensures participants’ privacy and accountability. The authors conclude that the BLOCIS framework can improve the effectiveness and efficiency of CTI sharing and enhance the overall security of the digital ecosystem. This study proposes a novel blockchain-based framework for sharing CTI in a Sybil-resistant manner, providing enhanced privacy and accountability. The BLOCIS framework could revolutionize the field of CTI sharing and contribute to a more secure digital ecosystem [22].

Borges Amaro et al. [23] designed a framework to address the challenges of managing the increasing amount of data generated by cyber threats and to provide organizations with a structured approach to making sense of the information. The proposed framework is comprised of six stages: data collection, data processing, data analysis, threat intelligence production, dissemination, and consumption. The study also discusses the various tools and techniques that can be used to implement each stage of the framework. Furthermore, the study emphasizes the importance of visualizing CTI data to aid in decision making and recommends the use of interactive dashboards and heat maps. The proposed methodological framework provides a comprehensive and structured approach to managing and utilizing CTI data for effective organizational decision making [23].

Al-Fawa’reh et al. [24] proposed a PCADNN model that combines principal component analysis (PCA) and deep neural network (DNN) algorithms to analyze network traffic data and detect anomalous behavior. The PCA algorithm was used to reduce the dimensionality of the input data, and the DNN algorithm was used to classify the data and identify abnormal patterns. The study’s authors conducted several experiments to evaluate the effectiveness of the proposed approach. The results showed that the PCADNN model could accurately detect anomalous network behavior with high precision and recall. Overall, the PCADNN model presented in this study provides a powerful tool for detecting cyber threats and improving organizations’ overall security posture. The method uses deep-learning algorithms to examine network activity data and find aberrant patterns, allowing enterprises to identify and react to cyber-attacks [24] rapidly.

In another study, Sun et al. [25] developed a technique based on automated intelligence production for cyber threat records using multi-source information fusion. The proposed approach integrates different data types, such as network traffic, system logs, and external threat intelligence feeds, to generate more comprehensive and accurate threat intelligence records. To show the viability of the suggested strategy, the authors created a prototype system. The study’s findings demonstrate that the technique may provide reliable threat intelligence records and boost the effectiveness of threat intelligence analysis. CTI has access to a wide range of data sources, including social media, the dark web, and open-source information, and it can analyze and keep track of these to gain vital insights regarding future cyber-attacks and their techniques. It enables businesses to actively reduce risks and safeguard their resources, systems, and networks. Moreover, CTI can provide situational awareness by informing organizations about emerging cyber threats and trends. It enables organizations to stay ahead of cyber attackers and respond more effectively to potential cyber incidents. The main challenges related to CTI include data quality, data overload, lack of standardization, and skill shortages. Thus, it can be challenging for organizations to build and maintain a CTI capability in-house. Implementing CTI can be expensive, particularly for smaller organizations with limited resources, which can make it difficult for them to justify the investment in CTI [25].

Serketzis et al. [26] highlighted organizations’ need for an effective incident response plan to deal with potential cyber-attacks. The authors also provide insights into how digital forensics can detect and investigate cyber-attacks and how actionable threat intelligence can improve the accuracy and speed of digital forensics investigations. The paper highlights the challenges faced by organizations in collecting and analyzing threat intelligence and provides recommendations for overcoming these challenges to achieve practical digital forensics measures [26].

Raptis et al. [27] highlighted that Connected and Autonomous Vehicles (CAVs) are becoming increasingly common, and thus cyber-attacks targeting them are also increasing. The authors developed a machine-learning-based framework called CAVeCTIR to address this issue. This framework utilizes natural language processing techniques and semantic analysis to match CTIRs with high accuracy. CAVeCTIR also includes a new feature selection method called “Minimum Redundancy Maximum Relevance” to select the most relevant features for matching CTIRs. The authors evaluated the performance of CAVeCTIR using a dataset of real-world CTIRs and compared it with two existing methods. The results showed that CAVeCTIR outperformed the current techniques, achieving an accuracy of 87.67%. The authors also conducted a sensitivity analysis and demonstrated the robustness of CAVeCTIR against different parameter settings. In conclusion, CAVeCTIR provides an effective solution for matching CTIRs related to CAVs, which can improve threat detection and response in the CAV ecosystem. The research shows the promise of machine-learning-based techniques to solve cybersecurity concerns in developing technology [27].

In another study, Alsaedi et al. [28] enhanced the accuracy of identifying harmful Uniform Resource Locators (URLs) by creating a detection model that uses CTI and two-stage ensemble learning. The model utilizes attributes extracted from internet searches and features based on CTI to enhance detection performance. The model proposed in this study demonstrates better results than other detection models, with an accuracy improvement of 7.8% and a reduction of 6.7% in false-positive rates compared to conventional URL-based models [28]. On the other hand, Van Haastrecht [29] highlighted that sharing platforms like the Malware Information Sharing Platform (MISP) could be helpful for SMEs if the shared intelligence is actionable. Therefore, a prototype application was developed to process MISP data, prioritize cybersecurity threats for SMEs, and provide customized recommendations. Further evaluations will refine the application and help SMEs to defend themselves against cyber-attacks more effectively [29]. Zhang et al. [30] proposed a solution called the CTI Automated Assessment Model (TIAM). TIAM evaluates sparsely populated threat intelligence from multiple perspectives. It utilizes automatic classification through feature extraction and integrates Adversarial Tactics, Techniques, and Common Knowledge (ATT&CK) to recognize attack methods linked with an Indicator of Compromise or IOC. The experiment demonstrates that TIAM could assess threat intelligence more efficiently, offering security managers valuable CTI [30].

In a study by Mishra et al. [31], anomalies in IoT networks were detected using message queuing telemetry transport (MQTT) and machine-learning algorithms, with a dataset of 4998 records and 34 features. Among the various classifiers employed, the random forest classifier demonstrated the highest level of accuracy at 99.94% [31].

The importance of sharing and promptly acting on high-quality CTI with the appropriate stakeholders is vital. To achieve this, Chatziamanetoglou and Rantos [32] proposed a blockchain-based system architecture for CTI that captures, evaluates, stores, and shares CTI while assessing its quality against predefined standards. The suggested system chooses validators and rates CTI inputs using a reputation- and trust-based method. The data are stored in a secure ledger that includes objective evaluation and validator performance and can be used to assess the reputation of CTI sources. The system’s dependability, consistency, and resistance to malicious activities were evaluated through a theoretical analysis using a probabilistic simulation, demonstrating an acceptable tolerance against malicious validators [32].

To overcome network security challenges, Li et al. [33] proposed an automatic CTI analysis method called K-CTIAA, which utilizes pre-trained models and knowledge graphs to identify threat actions from unstructured CTI data. K-CTIAA lessens the negative impacts of knowledge insertion, maps associated countermeasures using digital artifacts, and adds related knowledge in knowledge graphs to the corresponding place in CTI. In testing, K-CTIAA achieved an F1 score of 0.941 [33].

Sharing and exchanging CTI through blockchain technology can enhance protection measures, but existing models are susceptible to attacks and false reporting. Zhang et al. [34] proposed a novel blockchain-based CTI paradigm that integrates consortium blockchain and distributed reputation management systems for automated analysis and reaction to threat intelligence to overcome these problems. “Proof-of-Reputation” (PoR) consensus, a novel consensus method, satisfies the demand for a high transaction rate while establishing a reputation model for reliable network consensus. Experimental testing of the suggested model and consensus process revealed that it is safe and effective [34].

Data quantities have increased even more because of the proliferation of security devices and the growing complexity of information technology, causing difficulties for digital forensics and information security regulations. To solve this, Serketzis et al. [35] introduced the Digital Forensic Readiness (DFR) paradigm, which previously separated the concepts of forensic preparation and CTI. The model, which has good accuracy, precision, and recall rates and requires less data for analysis by researchers, is evaluated through experiments. The study indicates the value of integrating CTI and digital forensics procedures, offering a productive way to enhance operational DFR [35]. In addition, we have summarized the main contributions of each study. Table 1 summarizes the primary information from all papers relevant to detection model.

### 3.2. Knowledge Sharing and Training

In the context of knowledge sharing and training, Afzaliseresht et al. [36] discussed a common problem in cybersecurity in which organizations receive many machine-generated threat alerts. Still, only a very small percentage of them are investigated due to limited resources. To address this issue, they proposed a model that generates reports in natural language using storytelling techniques from security logs. This means that instead of receiving technical alerts that can be difficult for non-experts to understand, organizations would receive reports written in plain language with relevant information about potential threats and vulnerabilities. These reports can also be adjusted based on the reader’s level of expertise and preference. The proposed model is validated through a case study at a university’s Security Operations Center (SOC) and shown to provide better comprehension and completeness compared to existing methods for interpreting potential threats in cybersecurity contexts [36].

Sharing cyber threat information (CTI) is vital for enhancing security, yet many individuals are unwilling to share their CTI and prefer to merely consume it. In a research contribution, Riesco et al. [37] proposed a new approach to encourage sharing of CTI among several parties involved in cybersecurity information exchange. They suggest using blockchain technology and smart contracts to create incentives for knowledge sharing, which could contribute toward developing and implementing dynamic risk management systems to keep risks under control over time. Specifically, they suggest creating a marketplace for Ethereum blockchain smart contracts where participants can exchange CTI tokens as digital assets with a good value in the market. This would incentivize all parties to share their CTI while highlighting potential storage limitations/costs associated with transactions through simulations/experiments. Overall, this approach aims to improve security by encouraging more effective collaboration between stakeholders while navigating legal/regulatory constraints related to information sharing [37].

CTI focuses primarily on defense against these attacks, but there is a need for new methods to unmask attackers. Rana et al. [38] created malicious files as decoys, allowing the authors to gather information from susceptible PCs using honeypots. They used various tools for data analysis, including Visual Studio Code and Python. The evaluation method uses counterintelligence techniques such as cyber deception and decoy files to obtain adversary information. Overall, this research focuses on providing better proactive adversarial system intelligence by capturing attackers’ system information through accurate document-based tokens in a proactive defensive environment while executing threat hunting with TTPs (Tactics Techniques Procedures) [38].

In addition, Samtani et al. [39] developed a new system called the AZSecure Hacker Assets Portal (HAP), which is helps organizations increase their awareness of potential cyber threats by collecting data from various dark web platforms such as hacker forums, carding shops, Internet Relay Chat channels, and DarkNet marketplaces. The HAP platform uses advanced techniques such as CTI, data mining, and text mining to organize this information into an interface that allows for easy browsing, searching, and downloading content. It also offers dynamic visualizations to help scholars gain situational awareness and formulate novel research inquiries on emerging threat detection or critical hacker identification. HAP presently serves over 200 customers from academic institutions, police enforcement agencies, and industrial groups like General Electric and PayPal globally [39].

Koloveas et al. [40] presented a crawler architecture to collect cyber threat intelligence related to the Internet of Things (IoT) from the clear, social, and dark web. The architecture consists of four main components: a crawler module, a data pre-processing module, a data storage module, and a user interface module. The crawler module systematically and efficiently collects data from various sources, and the data pre-processing module cleans and filters the data. The data storage module securely stores the collected and pre-processed data and is scalable, and the user interface module provides a user-friendly interface for accessing the collected data. The proposed architecture was evaluated using a dataset of IoT-related cyber threat intelligence, and the evaluation results showed that the architecture effectively collects and processes cyber threat intelligence related to IoT from the clear, social, and dark web. Security analysts and researchers can use the proposed architecture to identify and analyze IoT threats and develop effective countermeasures to protect IoT devices and networks [40].

Basheer and Alkhatib [41] wrote a review article to outline the importance of testing and checking the dark web for CTI to prevent crimes and gain insight into criminal activities. The review includes recent research in the area, examining techniques, tools, methods, approaches, and outcomes. It also addresses technical hurdles, ethical considerations, and potential future developments [41].

Mundt and Baier [42] described an adaptive approach that uses CTI from the MITRE ATT&CK framework to simulate potential threats and identify weaknesses before they can be exploited. The process involves two main steps: automatically deriving the most critical threats for a business via CTI and designing a simulation gear based on attacks extracted from the MITRE ATT&CK framework to assess their impact. The aim is to enable companies to take proactive measures against data theft and double extortion attacks by simulating harmful technologies before they occur in operational environments [42].

On the other hand, Sakellariou et al. [43] emphasized using discussion forums as the raw data source for CTI and suggested a semantic schema for organizing the gathered data. The paper introduces the SECDFAN system, a comprehensive method for generating CTI products by analyzing forum content. Furthermore, a reference architecture was created systematically to address all CTI-related concerns, including product sharing and collaboration among security experts [43].

Sacher-Boldewin and Leverett [44] improved cyber defense by systematically categorizing and documenting possible failure states in a company’s security operations process. The system divides vulnerability management into three parts, starting from when a vulnerability is detected and rated as relevant, then asking whether prevention measures can be activated on time or if any signs of exploitation can be detected. The authors recommend using dimensions related to business processes, such as people, products, partners, etc. These dimensions are used to calculate the possible resolution categories by multiplying them by involved parties. The suggested system also highlights a direct connection between cybersecurity and risk quantification so that external and internal risks can be managed effectively and efficiently. It recommends building feedback options into existing processes by systematically categorizing possible failure states to help optimize workflows while delivering valuable metrics [44].

Koloveas et al. [45] introduced the “Integrated Framework for Threat Intelligence Mining and Extraction” system, INTIME. INTIME is a framework based on machine learning that gives a holistic perspective of the cyber threat intelligence process. It enables security experts to collect, evaluate, and exchange cyber threat data from various online sources, such as clear/deep/dark websites, forums, and social networks. Vulnerabilities/exploits/threat actors/cyber crime tools are among the information retrieved and managed via an integrated platform called MISP (Malware Information Sharing Platform), designed specifically for storing/sharing threat-related information across different organizations. One of the critical features of INTIME is its ability to gather CTI not only from structured sources like known security databases but also unstructured ones like deep net [45].

The importance of risk management in organizations is crucial, and real-time security threats can harm risk exposure levels. Riesco and Villagrá [46] highlighted companies’ challenges in managing risks, such as emerging techniques, asset complexity, and numerous vulnerabilities. To overcome these challenges, the authors suggest an architecture for dynamic risk assessment and management based on Web Ontology Language and Semantic Web Rule Language. The architecture includes a new semantic version of Structured Threat Information eXpression (STIX)v2.0 for exchanging CTI. The article demonstrates the effectiveness of the proposed framework in supporting decision making across different organizational levels using a leading cybersecurity organization. The proposed model aims to enable real-time risk management while integrating a mix of standards and ensuring ease of adoption [46].

Aljuhami and Bamasoud [47] investigated how Cyber Threat Information (CTI) can reduce cyber risks in Saudi universities by improving risk management. The study investigates CTI concepts, challenges, and risk management practices in higher education. Their work includes a review of previous studies and their relevance to the current research. The results highlight the importance of obtaining advanced and detailed information on cyber threats, or CTI, to deal with their constantly evolving nature. Integrating CTI into risk management enhances defenders’ ability to mitigate the increasing risk of cyber threats [47]. Sakellariou et al. [48] introduced essential CTI concepts and an eight-layer CTI in a similar study, with a reference model that can aid in the development of CTI systems. The model’s effectiveness is demonstrated through three case studies, resulting in the creation of CTI [48].

Dulaunoy et al. [49] developed a system for unreceptive DNS, malware hash archives, and Secure Sockets Layer (SSL) notaries. This system aims to support incident inquiries and infrastructure tracking by providing CTI. The authors explain that CSIRTs (Computer Security Incident Response Teams) use passive DNS and SSL databases to help with the incident reply. Still, they argue that their new passive SSH database would be a valuable addition to the CSIRT toolbox, because OpenSSH implementation is widely used on many servers as well as computers like MacOSs or Windows machines, which makes it an attractive target for attackers looking for vectors of attack or command-and-control mechanisms [49].

On the other hand, Gao et al. [50] developed a system called SecurityKG, which is a system that automates the collection and management of open-source CTI (OSCTI) from over 40 major security websites. It uses AI/NLP techniques to extract relevant information, such as potential threats, vulnerabilities, and risks to critical assets. The system also has an extendable backend that handles all gathered, extracted, and constructed OSCTI components. Additionally, it provides various interactivities through its user interface to facilitate knowledge graph exploration. SecurityKG aims to provide more comprehensive and accurate information about cyber threats [50].

Al-Mohannadi et al. [51] analyzed different types of web services and the ways in which adversaries can use them for malicious activities. The study suggests that CTI can be used to protect organizations from cyber threats by providing relevant information about potential attacks, vulnerabilities, and threat actors. This information can help organizations to develop better security strategies and responses to mitigate risks. The study highlights some of the main challenges related to CTI, such as the lack of standardization, data quality issues, and the need for skilled personnel to analyze and interpret the data. The research suggests that cloud-based web services can help to overcome some of these challenges by providing scalable and flexible solutions that can be customized to meet the specific needs of different organizations. The study suggests that cloud-based web services can enhance CTI by providing more comprehensive and accurate information about adversary activities. However, addressing the challenges related to data quality, standardization, and personnel skills is crucial to effectively use CTI to protect organizations from cyber threats [51]. Using AI and NLP to evaluate social media postings on cyber-attacks and electronic warfare, Sufi [52] offers a contemporary methodology. A single index is created for each nation using keyword-based index production techniques, and CNN is used to find abnormalities and their causes inside the index. The method is verified using real-time Twitter feeds, producing 75 daily cyber danger indices for six nations with anomalies. Decision makers may use the gathered intelligence to modify their cybersecurity readiness and lessen the harm done by cybercriminals [52].

In a different study, Cristea [53] examined the risks connected to potential threats from disruptive technologies in the context of the present financial systems. The study shows that by enhancing efficiency, cutting costs, and boosting transparency, disruptive technologies like blockchain, cryptocurrencies, and artificial intelligence have the potential to completely change the financial sector. However, these technologies also pose significant risks to the current financial systems, including cybersecurity threats, regulatory challenges, and financial instability. The study identifies five key categories of risks associated with disruptive technologies in the financial sector: technology, regulatory, market, operational, and systemic risks. The study concludes that though disruptive technologies significantly benefit the financial industry, managing the associated risks effectively is crucial. Financial institutions, policymakers, and regulators must work together to develop robust risk management strategies and regulatory frameworks to ensure that the benefits of disruptive technologies are realized while mitigating their potential risks [53].

Thach et al. [54] suggest that Industry 4.0 has greatly impacted the banking industry in Vietnam, especially regarding its technology quality management practices and cybersecurity risk management strategies. The paper identifies several key factors affecting the success of Industry 4.0 implementation in the banking sector, including regulatory compliance, data privacy, and talent management. Furthermore, the study highlights the importance of cybersecurity risk management in the banking industry, given the increasing prevalence of cyber threats and attacks. In the context of Industry 4.0, the study offers a thorough methodology for managing cybersecurity risks that includes proactive risk identification, risk assessment, risk reduction, and risk monitoring. Overall, the research underlines the necessity for financial institutions in developing nations like Vietnam to establish strong practices for technology quality management and cybersecurity risk management in order to be secure and competitive in the quickly changing digital environment of Industry 4.0 [54].

Tripodi [55] utilized a sociotechnical framework to analyze the sociopolitical and technological factors contributing to misinformation’s spread and persistence. They argue that the continuation of this misinformation is due to a complex interplay of social and technological factors, including political polarization, the use of social media platforms to spread misinformation, and the amplification of misinformation by influential individuals and organizations. The study also highlights the potential public health consequences of the persistence of this misinformation, including increased transmission of viruses and decreased compliance with public health guidelines. The authors concluded that addressing this issue will require a multifaceted approach that considers the social and technological factors contributing to the spread of misinformation [55].

Odemis et al. [56] developed a “Honeypsy” system designed to observe user behavior with CTI. The system collects and analyzes data from honeypots and decoy systems that mimic natural systems and are used to detect and monitor cyber threats. Honeypsy analyzes the gathered data and looks for unusual behavior using machine-learning methods. The system is appropriate for business situations, since it was made to be scalable and manage large volumes of data. The authors of the research carried out many tests to assess Honeypsy’s performance in identifying user activity in CTI. The results showed that the system can accurately detect anomalous behavior, such as the use of malicious tools and techniques, and can provide early warnings of potential cyber-attacks. The Honeypsy system offers a promising approach to detecting user behavior using machine-learning algorithms and honeypot data in CTI. The system can potentially improve the accuracy and efficiency of cyber threat detection in enterprise environments, helping organizations better protect their assets and data from cyber-attacks [56].

Vevera et al. [57] proposed an approach to help organizations make informed decisions when selecting CTI solutions and ensure they align with their specific needs and requirements. The proposed approach is comprised of six attributes: accuracy, reliability, timeliness, cost-effectiveness, usability, and comprehensiveness. This study thoroughly explains each attribute and discusses the criteria that can be used to evaluate them. Furthermore, the study’s authors conducted several experiments to demonstrate the effectiveness of the recommended technique. The findings suggest that the multi-attribute strategy can effectively evaluate CTI products and services and help organizations make informed decisions when selecting solutions that best fit their needs and requirements. Overall, the multi-attribute approach proposed in this study provides a structured and comprehensive approach to selecting CTI products and services. The system can help organizations to evaluate and compare different solutions based on multiple criteria, thereby improving the quality of their decision-making process and enhancing their overall cybersecurity posture [57].

In another study, Du et al. [58] highlighted the significance of CTI sharing in mitigating the risk of cyber-attacks and discussed the challenges and obstacles that hinder the sharing process. Moreover, the study presents the key players and initiatives involved in CTI sharing, such as the government, the private sector, and international organizations. Finally, the study provides an outlook for CTI sharing, with recommendations for enhancing the process and increasing its effectiveness [58].

Westerlund [59] discusses the potential implications of this technology for business and society, including the risks of cyber threats, such as fraud, identity theft, and misinformation. The author also examines the various applications of deep fake technology, such as entertainment and politics, and discusses the challenges of regulating and controlling its use. Overall, the article provides a valuable overview of the emergence of deep fake technology and its potential impact on business and society [59].

Due to privacy issues and the absence of a common dataset format, developing a machine-learning-based detection system using heterogeneous network data samples from different sources and organizations is challenging. Sarhan et al. [60] presented a cooperative CTI-sharing system that enables several enterprises to create, train, and test a powerful ML-based network intrusion detection system to solve this issue. A federated learning method was recommended to protect the confidentiality of each organization’s data. Additionally, network data traffic was made accessible in a common format to help uncover significant trends across various data sources. The authors used NF-UNSW-NB15-v2 and NF-BoT-IoT-v2, among other datasets and scenarios, to test the proposed framework. They found that it correctly classifies various traffic kinds without the necessity for inter-organizational data sharing.

To promote the standardized exchange of cyber threat intelligence, industry standards, including STIX, Trusted Automated Exchange of Intelligence Information (TAXII), and Cyber Observable eXpression (CybOX), were developed. Ramsdale et al. [61] evaluated these standards. The writers examine the formats and languages that can be used to exchange cyber threat intelligence as well as publicly available sources for threat feeds. Additionally, they look into the types of data offered by a sample of cyber threat intelligence feeds and the challenges involved in compiling and distributing data. The writers matched the data type and the data needs for various security activities. They conclude that many standards are poorly adopted and implemented, with suppliers preferring unique or conventional forms [61].

Oosthoek and Doerr [62] highlighted the inadequacy of the CTI (Cyber Threat Intelligence) field in their analysis; this is primarily due to flawed methodology. As a result, CTI currently delivers ineffective outcomes. However, the field has the potential to mature and improve by drawing from the methodology of its parent field, intelligence studies, and addressing challenges related to quality, bias, and actor naming. The article emphasizes the importance of scientific scrutiny and suggests that an alliance between the Intelligence Community (IC) and CTI can drive cyber defense in the future [62].

De Melo e Silva et al. [63] discuss the need for more efficient defense mechanisms in response to the changing cybersecurity landscape and emerging threats. They provide an overview of the cyber threat intelligence scenario and identify relevant standards and platforms. The evaluation of these standards and platforms reveals that STIX is the most widely adopted standard due to its holistic approach. At the same time, the Malware Information Sharing Platform (MISP) and OpenCTI are considered the most comprehensive and flexible platforms. However, finding a comprehensive solution for defense based on threat intelligence remains a challenge due to the divergent focuses of existing platforms [63]. Table 2 provides an overview of studies focusing on knowledge sharing and training.

## 4. Discussion

**Figure 3:**
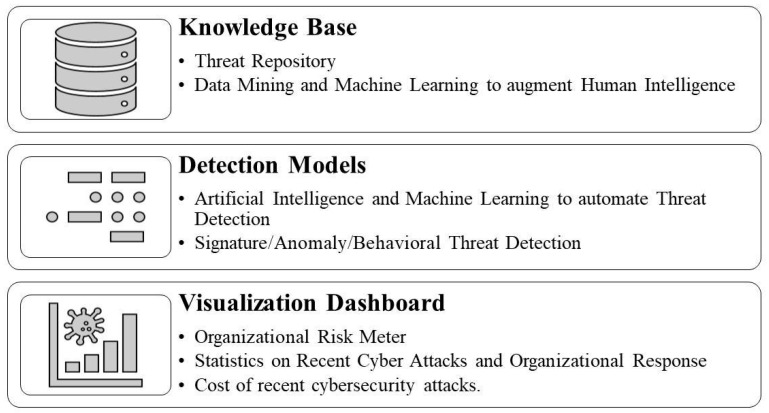
Proposed layered CTI framework.

Based on the literature review, developing a sophisticated and comprehensive CTI framework is crucial for organizations to manage and mitigate potential risks effectively. Cyber threats are becoming increasingly complex, making it difficult for traditional security measures to keep up. A well-designed cybersecurity threat intelligence framework can help organizations to avoid emerging threats by providing real-time insights into their critical assets’ potential risks. Such a framework should include identifying vulnerabilities within an organization’s infrastructure, detecting anomalous behavior or patterns associated with malicious activity, and sharing information about potential threats with stakeholders. Furthermore, having a structured approach toward cybersecurity threat intelligence helps to ensure consistency across different organizational departments. It also enables better collaboration between teams who are responsible for managing cyber-attack risk exposure. Accordingly, we propose a CTI framework to improve business organizations’ cyber threat response capabilities in this section. Figure 3 shows the architecture of the security framework presented in this paper. The proposed framework comprises a knowledge base, detection models, and visualization dashboards.

The first layer, the knowledge base, includes information about potential threats, vulnerabilities, and risks to critical assets. This information can be obtained from sources like internal logs, the dark web, or external feeds. Organizations should establish a centralized repository of this information that all organizational stakeholders can access. This will help ensure that everyone has access to up-to-date threat intelligence data and can make informed decisions regarding risk management. Furthermore, machine-learning algorithms are increasingly used to analyze large volumes of data to identify patterns associated with cyber-attacks or malicious activity. By leveraging these technologies along with human expertise, organizations can improve their ability to develop a reliable database quickly and effectively. A comprehensive knowledge base is crucial for practical cybersecurity threat intelligence, providing valuable insights into potential risks aimed at an organization’s critical assets.

Human identification of cyber threats is limited due to the cognitive limitations of humans., As highlighted in the literature [12,25,27,31,45], machine-learning and artificial intelligence tools can help to identify malicious network traffic. Therefore, the proposed detection models’ second layer includes applications that include these technologies for signature-based, anomaly-based, and behavior-based detection. Signature-based detection involves comparing incoming traffic against known signatures or patterns associated with previously identified threats. This approach could prove to be effective at detecting known threats but may not be able to detect new or emerging threats. Anomaly-based detection involves identifying deviations from regular network activity that could indicate potential malicious activity. This approach could help to detect unknown attacks but may also generate false positives if legitimate activities are flagged as abnormal. Behavioral-based detection focuses on monitoring user behavior and identifying unusual actions deviating from established norms. This model could help to identify insider threat actors with authorized access to systems. Combining these approaches and machine-learning algorithms can help to improve accuracy and reduce false positives/negatives. By leveraging multiple layers of protection, organizations can quickly enhance their ability to detect emerging cyber threats while minimizing risk exposure.

The third layer, the visualization dashboard, provides an overview of key metrics related to cyber threats, such as the number of attacks detected, as well as types of attacks and their severity level. As shown by Samtani et al. [39], the visualization of cyber threats increases situational awareness among actors, so visualization tools can be used to represent complex data sets in a more intuitive way, which helps analysts to identify patterns or trends that may not be immediately apparent from raw data. This approach is advantageous when dealing with large volumes or diverse sources of information. The dashboards should be customizable based on user roles so stakeholders within an organization can access relevant information quickly. Visualization tools such as heat maps, graphs, etc. could help analysts to understand how different events are connected and their impact on critical assets. They can also be used to highlight which infrastructure assets will have higher vulnerabilities or how cybersecurity risks vary based on the current organizational situation. Overall, dashboards and visualization tools enhance cybersecurity threat intelligence by providing real-time insights into organizational data, such as an organizational risk meter, showing potential risks aimed at an organization’s critical assets, the number of recent attacks, threat levels, the average organizational response time, and the cost of recent cybersecurity attacks. By utilizing these technologies, organizations can quickly improve their ability to detect emerging threats while minimizing risk exposure.

Due to the enhanced digital transformation, business organizations must be resilient toward cybersecurity [64,65,66,67]. Given the increasing frequency and sophistication of cyber-attacks today, developing a robust cybersecurity threat intelligence framework should be considered one of the top priorities of any organization looking to protect its critical assets. Since humans are a weak link in cybersecurity [68,69,70], a robust CTI framework will also facilitate overcoming human security lapses. As proposed in our model, leveraging multiple layers such as a knowledge base, detection models, and visualization tools along with human expertise would enable effective management and mitigation of these evolving challenges.

Based on the details presented in this paper, there are a few limitations to consider. First, the proposed framework for CTI implementation may not apply to all organizations, as each organization has unique needs and resources. Therefore, organizations need to tailor their approach based on their specific requirements. Second, collaboration between stakeholders is essential for practical CTI; it can also pose challenges, such as restrictions on information sharing due to legal or regulatory constraints [71,72]. Organizations must navigate these challenges carefully while complying with relevant laws and regulations. Third, the empirical analysis conducted in this study was limited by the sample size and the scope of data collected from selected industries. This may limit its generalizability across other sectors or regions globally where CTI programs have been implemented successfully but were not included within this research project’s scope of work. Finally, and importantly, cyber threats continue to evolve rapidly over time, making it difficult, and even sometimes impossible, to keep up with new emerging trends without continuous monitoring and updating of one’s security posture strategy plan accordingly. Therefore, any framework should be considered dynamic rather than static when implementing CTI frameworks within an organization’s security posture strategy plan.

## 5. Conclusions

In conclusion, this paper significantly contributes to the field of CTI by proposing a comprehensive framework for the implementation of CTI. Through an extensive literature review, key components that are essential for practical CTI have been identified, including data collection and processing, analysis, and dissemination. The proposed framework could provide valuable guidance for organizations seeking to establish or improve their CTI capabilities. Additionally, the methodology employed in this research can serve as a model for future studies on related topics. In addition, the paper’s emphasis on collaboration between different stakeholders is particularly noteworthy, as it highlights the importance of information sharing in combating cyber threats. By bringing together experts from various fields, such as cybersecurity professionals, law enforcement agencies, and government officials, organizations can quickly leverage their collective knowledge to identify emerging threats. Several areas for future work could build upon the findings of this study. First, further research could be conducted to explore how organizations can effectively tailor their approach to CTI implementation based on their specific needs and resources. Second, additional studies could investigate how collaboration between stakeholders can be facilitated more effectively while navigating legal and regulatory constraints related to information sharing.

## Figures and Tables

**Table 1:** Summary of papers related to the detection model.

Ref. No.	Publication Year	Main Contribution	Technology/Method	Source of Data
[12]	2022	The paper develops a model based on statistical characteristics to detect DGA-based traffic and explores the application of artificial intelligence/machine learning (AI/ML) in CTI.	Random forest algorithm (ML)	DNS query logs from a campus network
[13]	2022	The authors suggest a unique multimodal classification method based on understandable deep learning that categorizes onion services depending on their picture and text content.	Gradient-weighted Class Activation Mapping Convolutional Neural Network with a trained word-embedding algorithm with additive attention from Bahdanau	Dark web onion service images and texts
[14]	2022	The paper describes how unstructured CTI data may be used to gather cyber threat intelligence. The authors developed a novel model called “Attack2vec” that outperforms other models. The detailed feature set used in the model TTP tools, target company, virus, and application are all included. The usage of a comprehensive feature set improves classification outcomes.	Novel Attack2vec embedding model	Unstructured cyber threat intelligence reports
[15]	2021	In the research, an EX-Action framework for automatically extracting threat actions from natural language processing (NLP) technologies and a multimodal learning algorithm for creating CTI reports are developed. Utilizing a measure, the extracted activities were assessed for information completeness. The efficiency of the framework is compared to that of two state-of-the-art action extraction methods in terms of precision, recall, accuracy, and F1 score. In order to better defend against network threats, intelligence-based active defense sharing was improved.	NLP and multimodal learning algorithms	CTI reports consisting of sentences with complex structure
[16]	2020	The authors propose a blockchain-based intelligence on cyber threat system architecture for long-term computing to handle dependability, confidentiality, scalability, and sustainability challenges in collecting and analyzing data to identify potential threats. The model was proposed to work with multiple feeds, provide a trustworthy dataset, minimize network congestion, and stimulate participation by quantifying companies’ contributions. Additionally, through experimental study, the proposed model’s success was assessed using various metrics, including dependability, privacy, scalability, and sustainability.	NLP and multimodal learning algorithms	IP information, domains, URLs, network artifacts, and aggregation
[28]	2022	In order to build a cyber threat intelligence-based detection model, the article addresses a study that intends to improve the identification of hazardous URLs by applying two-stage ensemble learning. The suggested approach outperformed detection methods from prior research, improving accuracy by 7.8% and reducing false-positive rates by 6.7% when compared to conventional URL-based models.	Customized algorithm	Scholarly journal- malicious URL
[29]	2023	The report offers a systematic evaluation that contends that SMEs may profit from threat information-sharing platforms like MISP if shared intelligence is transformed into useful insights. In order to evaluate MISP data, rank cybersecurity hazards for SMEs, and provide personalized advice, a prototype application is developed.	-	-
[30]	2022	The authors of this paper used automatic classification based on feature extraction and integrated ATT&CK to identify attack methods associated with IOC.	CTI systems modeling	Community-sourced threat intelligence and open-source intelligence
[31]	2022	This paper focuses on potential network attack identification, and countermeasures are recommended utilizing simulated data. Anomalies in IoT networks are detected using message queuing telemetry transport (MQTT) and machine-learning algorithms.	Mixed methods using quantitative and qualitative approaches	Al-Kasassbeh dataset
[32]	2023	In this paper, the authors underscore the significance of acquiring advanced and in-depth information about cyber threats in Saudi universities.	Probabilistic approach	GitHub repository
[33]	2023	In this paper, the authors propose an automatic CTI analysis method called K-CTIAA to address the challenges of analyzing these threats. K-CTIAA pre-trained algorithms and knowledge graphs were used to obtain threat actions from unorganized CTI and achieved high automatic threat intelligence analysis performance.	K-CTIAA/BERT analysis	An open-source APT
[34]	2018	The authors of this paper present a collaborative cyber threat intelligence-sharing scheme to allow many enterprises to collaborate on the design, training, and evaluation of a powerful ML-based network intrusion detection system.	Consortium blockchain	CTI data
[35]	2019	The paper proposes a DFR model that combines CTI and forensic preparedness to help increase Digital Forensics Readiness and minimize the time and expense of response to incidents and investigations. The model achieved high accuracy, precision, and recall rates while reducing the amount of information that investigators must study, demonstrating the effectiveness of combining CTI and digital forensics processes.	Digital Forensic Readiness	Local log dataset
[27]	2022	This paper presents a unique technique for detecting commonalities amongst CTI reports describing harmful actions identified on CAVs. This unique model achieved 96% accuracy, 96.5% precision, 95.58% recall, and 95.75% F1 score, respectively.	Decision Tree, Random Forest, and Support Vector Machine are examples of machine-learning models.	Reports from the scientific community, security manufacturers, and a programmable Google search engine
[22]	2020	In this study, the authors created a blockchain-based CTI framework that can swiftly identify and reject false data in order to defend against a Sybil attack and increase confidence in the source and content of data. The suggested architecture collects CTI via a process certified by smart contracts and stores data meta-information on a blockchain network.	Blockchain-based open architecture for exchanging cyber threat intelligence (BLOCIS)	Utilizes open-sourced intelligence as a route for data acquisition
[23]	2022	The authors of this research created a platform to address situations in which a cybersecurity analyst may import threat data, analyze it, and generate a timeline to gain insight and properly contextualize a threat. The results demonstrate that knowledge is facilitated about the environment in which the threats are placed, making vulnerability mitigation more effective.	Timeline representation of danger details and analytical data insights	Multiple sources
[17]	2021	The methodology used in this study examines potential danger indications gathered by smart meters and proposes a method for acquiring cyber threat information that focuses on the energy cloud. Through a mechanism for exchanging and distributing knowledge about cyber threats across the Advanced Metering Infrastructure (AMI) and cloud tiers, this research also provides a method for quickly deploying a security framework to a significant energy cloud architecture.	A setting that models an attacker model and an energy cloud system	Every second, threats employing energy item data and 20,480 IoC data instances are broadcast from a prosumer device.
[24]	2021	This paper enhances IDS detection mechanisms by incorporating novel features for identifying threats based on two assumptions related to handling zero-day attacks with constrained computing power and resources, as well as a comprehensive approach for detection by combining DNN and principal component analysis (PCA) to enhance security and performance.	The accuracy rate of DNN using PCA and model was 98%.	Initial packet capture (PCAP) is a common method for collecting network traffic data.
[18]	2022	In this paper, the authors highlight the use of effective visualizations for CTI. A preliminary analysis of the data of CTI reports was carried out to unearth and depict relevant cyber threat trends, allowing security professionals to reduce vulnerabilities and proactively forecast cyber-attacks in their networks.	Based on machine-learning approaches, a system for visually analyzing CTI data is developed.	TTP dataset
[19]	2021	This article demonstrates a proof of concept (PoC) using blockchain technology to secure private networks, Internet of Things (IoT) devices, and internet service providers (ISPs). The findings back up the idea of decentralized cyber threat intelligence-sharing networks that are capable of protecting several stakeholders.	A proof of concept (PoC) using blockchain technology to secure home networks, Internet of Things (IoT) devices, and internet service providers	Data collected at the ISP and the customer premises, equipment (CPE) routers
[20]	2022	This paper analyzes CTI VirusTotal (VT) large-scale field data. The authors discovered that the threat intelligence given by VT is inefficient, and the proposed method can improve CTI.	TriCTI is a trigger-enhanced system that discovers actionable threat intelligence, conveying a fuller context of IOCs by disclosing their campaign phases.	DS-1 (2013–2020) and DS-2 (2021) datasets are used.
[25]	2021	This article offers an automated technique to produce CTI records by merging NLP, neural networks, and cyber threat intelligence expertise.	A method for utilizing cybersecurity threat intelligence data together with NLP, machine learning, and CTI records is automatically generated based on multi-type OSTIPs (GCO).	GCO was performed on the collected OSTIPs, yielding 24,835 articles published between 2010 and 2019.
[26]	2018	In this paper, the authors highlight that CTI has become a common practice for preventing or detecting security incidents, especially in the digital forensics (DF) domain.	Creation of a unique methodology for boosting the efficacy of current digital forensic readiness (DFR) schemes by exploiting cyber threat information-sharing capabilities	Local IoC database
[21]	2017	This article presents a banking Trojan feature taxonomy based on a cyber death chain. This danger intelligence-based taxonomy, which provides stage-by-stage operational knowledge of a cyber attack, can help security practitioners as well as aid in the construction of evolving artificial intelligence for Trojan detection and mitigation strategies.	A taxonomy that provides operational knowledge of a cyber-attack stage by stage	127 financial transactions from the real world

**Table 2:** Summary of papers related to knowledge sharing and training.

Ref. No.	Publication Year	Main Contribution	Technology/Method	Source of Data
[36]	2019	Using storytelling approaches, the authors suggest a mechanism that creates insights into the natural language from security data.	Log-driven storytelling model using narrative techniques and human-centered data mining	Security logs
[38]	2022	The authors use novel methods for circumventing virtual private networks (VPN) and additional security measures to gather accurate source information.	The counterintelligence and counterattack approach employs an Elastic Sky X Integrated (ESXI) server in a data center, public and private pathways for accessing attacker logs, Cowrie and Windows honeypots with numerous open ports like Secure Shell (SSH) to confuse attackers, and a log server to store logs.	Attacker logs and Cowrie and Windows honeypots
[49]	2022	The main benefits of the system described in this article include the fact that it allows for the storage and retrieval of SSH connections used to collect historical forensic artifacts and provides a Representational State Transfer (REST) API to aid in incident investigations and infrastructure monitoring.	The program maintains fingerprints in a Redis-compatible backend and offers an API that uses REST to put information into a datastore and obtain signatures.	Server banners, key types, and IP addresses
[39]	2021	The primary goal of this study was to increase awareness of situations in cybersecurity by offering greater active inspection of possible dangers that are developing in cyberspace before an assault. The AZSecure Hacker Assets Portal (HAP) gathers, analyzes, and publishes on dark web data sources to provide a unique view of hackers and associated cybercriminal assets while adding CTI insights to increase awareness of the situation.	CTI, text, and data mining (key exchange algorithms, encryption algorithms, and message authentication code algorithms)	Dark web cyber-attack tools
[42]	2022	The primary goal of this work was to offer an idea to accomplish targeted automated data exfiltration mitigation along with a preliminary assessment. The authors propose using international approaches and the MITRE ATT&CK framework to automatically recognize and simulate the most relevant data exfiltration risks, strictly focusing on mitigating these threats.	Mapped Building Security in Maturity Model (BSIMM) and threat-based security concepts	Automatic procedures based on the framework would be network traffic and persistent data.
[50]	2021	This article describes SecurityKG, a system proposed to collect and manage open-source security threat intelligence (OSCTI) information. SecurityKG extracts high-fidelity information about threat behaviors using AI and machine learning and builds a security-knowing graph.	Artificial Intelligence and natural language processing techniques	OSCTI reports
[44]	2022	The major goal of this study was to demonstrate how false-positive occurrences can be identified methodically for all services and the ways in which this data can be utilized to suggest areas for development. The study also focuses on recognizing and recording issues that arise while detecting and analyzing vulnerabilities and whenever the security operations center (SOC) attempts to add an inventory source for continuous monitoring.	Systematic categorization of possible failure states and building these into existing security workflows and tools	False positives, incorrect states, and cyber defense operations issues
[37]	2019	The major goal of this work was to offer a novel CTI-sharing model that encourages all participants at all levels to communicate important information in real-time. The suggested solution uses the blockchain and guidelines such as Generalized Threat Information Transfer and World Wide Web Consortium (W3C) semantics web standards to allow for a workspace of information linked to behavioral threat intelligence patterns. This will aid in characterizing strategies, approaches, and processes while also rewarding CTI sharing via an Ethereum-based smart contract marketplace.	Ethereum blockchain smart contract marketplace	CTI data
[45]	2021	This article presents IN TIME, a machine-learning-based architecture that provides a complete platform for managing cyber threat intelligence. The framework may be used by security analysts to swiftly find, acquire, assess, extract, integrate, and distribute information on cyberthreats from diverse online sources. It also supports the whole threat lifecycle through open standards and user-friendly interfaces, allowing for the quick deployment of data collection services and the automatic grading of acquired information.	Machine-learning-based framework called IN TIME	Internet sources
[46]	2019	The article emphasizes the importance of risk management in organizations and the challenges they face in managing risks. It proposes a new architecture for dynamic risk assessment and management, which enables real-time risk management while ensuring ease of adoption by incorporating a mix of standards. The article demonstrates the effectiveness of the proposed framework in supporting decision making across different organizational levels using a leading cybersecurity organization.	Protegé	Semantic data model
[52]	2023	The paper covers the possible damage that cyber-attacks may do to the world economy and the necessity of comprehending the danger level to modify cybersecurity measures at various levels. It suggests a modern technique for analyzing the context of social media posts on cyber-attacks and electronic warfare using AI and NLP. Seventy-five daily cyber threat indices for six countries are produced due to the technique’s validation utilizing real-time Twitter feeds.	Twitter feed, AI, and NLP	Social media
[62]	2021	The article highlights the vulnerability of small- and medium-sized enterprises (SMEs) to cyber-attacks due to their lack of resources. It discusses the need to share CTI to assist SMEs in cybersecurity defense. However, existing shared intelligence approaches do not sufficiently meet SME requirements, and further investigation is necessary to enhance SME cybersecurity resilience. A prototype application was developed to process MISP data, prioritize cybersecurity threats for SMEs, and give customized recommendations. Future evaluations will refine the application and help SMEs to defend themselves against cyber-attacks more effectively.	The body of studies on intelligence draws on qualitative approaches used in social science. research.	Live social media feeds
[47]	2021	The paper explores how CTI can reduce cyber risks in Saudi universities by improving risk management. It examines CTI concepts, challenges, and risk management practices in higher education. It concludes that integrating CTI into risk management can enhance defenders’ capacity to mitigate the risk of cyber threats.	-	-
[48]	2022	In order to aid in the design of CTI systems, this study emphasizes a number of important CTI ideas and an eight-layer CTI reference model. A powerful ML-based network intrusion detection system may be designed, trained, and evaluated by several enterprises working together through a collaborative cyber threat intelligence-sharing scheme.	CTI model design methodology	Existing CTI platforms
[43]	2023	The authors of this paper propose a semantic schema for organizing collected data. The SECDFAN system is introduced as a comprehensive approach for creating CTI products by analyzing forum content.	SECDFAN’s CTI reference architecture	Repository data
[60]	2023	The authors present a strategy for collaborative cyber threat information sharing to allow several enterprises to collaborate on creating, training, and assessing an effective ML-based intrusion detection system.	Federated, centralized, and localized learning scenarios	Local data
[61]	2020	In this paper, researchers explore ways to assist SMEs in their cybersecurity defense through CTI sharing. Existing shared intelligence approaches do not meet SMEs’ requirements; further investigation is needed to enhance cybersecurity resilience.	Methods and platforms for sharing CTI	CTI data
[41]	2021	The article discusses the significance of analyzing dark web content for CTI to deter cybercrimes and understand criminal behavior.	The life cycle of CTI	Internal network data, external threat feeds, open-source intelligence (OSINT), human intelligence
[63]	2020	In this paper, the authors emphasize that shared intelligence needs to be translated into actionable insights to be effective. A prototype application is created to process MISP data, prioritize cybersecurity threats, and give customized recommendations.	5W3H method	Open-source TI platform
[56]	2022	The writers of this work want to highlight the nuances of hackers’ personalities and competence to assist defense specialists of targeted institutions in developing cybersecurity tactics based on the hackers’ modus operandi.	The relationship between a hacker’s behavior/logs in a server and the hacker’s personality, skills, and psychology	Logs
[57]	2022	A case study is investigated based on user assessments and reviews of security threat intelligence providers. The effect of VIseKriterijumska Optimizacija I Kompromisno Resenje (VIKOR) user parameter modification on CTI provider ranking is investigated. The suggested approach is a tool to help security program executives decide which CTI providers to use. It also assists CTI service providers in improving the quality of their goods and services.	Support tools for security program executives confronted with the issue of which CTI providers to choose. It also assists CTI service providers in improving the quality of their goods and services.	Security threat intelligence internet platform
[51]	2019	To enable active threat intelligence, this study provides a threat intelligence framework for evaluating attack data acquired via a cloud-based web service.	Analyzing attack data acquired via cloud-based web services to provide active threat intelligence	Data cloud
[58]	2020	The significance and value of threat intelligence are covered in the opening paragraphs of this article. A threat intelligence analysis model is then presented. Next, the study compiles and organizes the suppliers of threat intelligence as well as the threat intelligence-sharing policies.	The existing threat intelligence-sharing method has several major flaws.	-
[40]	2021	The writers concentrate on the task of acquiring information in this study. They demonstrate a unique crawling architecture for openly gathering data from clear web security websites, social web security forums, and dark web hacker forums/marketplaces. The suggested architecture divides data collection into two phases.	Information-gathering task	-
[59]	2019	The research includes a complete assessment of deep fakes and economic potential for cybersecurity and AI businesses battling multimedia fraud and fake news.	A comprehensive review of deep fakes	-
[53]	2021	This article focuses on integrating, comparing, and examining disruptive technologies’ effects, presenting security threats and occurrences, and building risk management strategies.	Design measures to manage risk	_
[54]	2020	As the important financial sector adapts to greater autonomy, there is a risk of increasing vulnerabilities and amplification of the impact of cybersecurity threats. As a result, companies must possess the flexibility to invest in ICT and cybersecurity expenditures to adjust to unanticipated conditions swiftly and efficiently for improved technology quality management.	Risk management plans	-
[55]	2022	This study provides a deeper knowledge of the main stories being shared by ReOpen members as well as the sources they use to back up their opinions. Members offered public safety solutions based on individualism and self-inquiry while trying to reinterpret data to reduce the danger of COVID. Members questioned the veracity of the fact checkers when the platform tried to uncover problematic content, highlighting the intimate connection between misinformation and epistemology.	It clarifies the primary narratives circulating among ReOpen members and the material they used to back up their claims.	Facebook groups

## Data Availability

Not applicable.
